# Comparing methods of adenosine administration in paroxysmal supraventricular tachycardia: a pilot randomized controlled trial

**DOI:** 10.1186/s12872-022-02464-5

**Published:** 2022-01-26

**Authors:** Phruek Daengbubpha, Borwon Wittayachamnankul, Krongkarn Sutham, Boriboon Chenthanakij, Theerapon Tangsuwanaruk

**Affiliations:** Department of Emergency Medicine, Faculty of Medicine, Chiang Mai University, 110 Inthawaroros Road, Sribhumi, Amphoe Muang Chiang Mai, Chiang Mai, 50200 Thailand

**Keywords:** Adenosine, Arrhythmias, Electrocardiography, Emergency department, Randomized controlled trial, Tachycardia

## Abstract

**Background:**

Intravenous adenosine is the recommended treatment for paroxysmal supraventricular tachycardia (PSVT). There is no official recommended method of giving adenosine. We compared the success rates between a standard and alternative method of first dose intravenous adenosine in PSVT.

**Methods:**

A pilot parallel randomized controlled study was conducted in the emergency department of a tertiary care hospital. Eligible patients were stable PSVT adult patients. We used block randomization and divided them into two groups, the standard method (double syringe technique of 6 mg of adenosine), and the alternative method (similar to the standard method, then immediately followed by elevating the arm to 90° perpendicular to a horizontal plane for 10 s). The primary outcome was the success rate of electrocardiogram (ECG) response which demonstrated termination of PSVT (at least two-fold of the RR-interval widening or sinus rhythm conversion). Secondary outcomes were complications within one minute after the injection.

**Results:**

We allocated 15 patients in each group and analyzed them as intention-to-treat. The success rate was 86.7% in the alternative group and 80% in the standard group (risk difference 6.7%, 95% confidence interval − 19.9 to 33.2%, *P* 1.00). Complications within one minute after adenosine injection were also similar in both groups, 14 of 15 patients (93%) in each group had no complications, without significant difference.

**Conclusions:**

No evidence of the difference between alternative and standard methods occurred, in terms of the success rate of ECG response and complications within one minute after adenosine injection. The standard method of adenosine injection is a safe, easy-to-administer, and widely available treatment for PSVT.

*Trial Registration*: TCTR20200609001.

## Introduction

Supraventricular tachycardia (SVT) is a common cardiac problem, which can be a life-threatening condition requiring immediate treatment, in the emergency department (ED). Paroxysmal supraventricular tachycardia (PSVT) is an abnormal fast cardiac rhythm, originated above the bundle of His, mostly atrioventricular (AV) node. SVT is defined as a regular narrow QRS-complex tachycardia (QRS interval < 120 ms [msec]). Its incidence about 1 in 500 in adult patients [1, 2].

Adenosine is an endogenous, rapidly metabolized, purine nucleoside [1]. Its action is transiently blocking myocardial conduction at the AV node, making it therapeutically useful for slow heart rate in SVT [1, 3, 4]. Moreover, due to its fast metabolization (approximate half-life is about 2–5 s), a fast intravenous injection is required for the best treatment outcome. The standard recommended treatment of acute hemodynamically stable SVT supported by the 2020 American Heart Association update on Advanced Cardiovascular Life Support (ACLS) is adenosine intravenous double syringe technique via a T connector. The starting and subsequent dosages are 6 and 12 mg intravenous route in adults, immediately followed by normal saline solution 20 mL [3, 4].

Over the past, many studies evaluated the efficacy, route of administration, safety, and recommended dosage use in treatment PSVT [5, 6]. Specifically, a prospective randomized study focused on the new mixed convenient method of intravenous adenosine (mixed adenosine and saline in the same syringe) for PSVT compared with the recommended standard method. The new method was significantly more convenient than the standard one, while the success rate was equal in both groups [7]. Some practice guidelines regard an alternative way of giving intravenous adenosine utilizing a double syringe technique in the antecubital vein followed by elevation of the arm. A video showing intravenous adenosine intravenous (ACLS Pharmacology) administration shows this method [8]. This alternative method has no proven literal evidence, no studies have mentioned its success rate as an alternative method for adenosine administration. This randomized controlled study will provide data on the success rate and complications of the alternative method compared to the standard method, which provide future evidence for PSVT treatment.

This study aimed to compare the success rate of first dose intravenous adenosine between the standard and alternative methods of intravenous adenosine in a patient with stable PSVT**.** We hypothesized that the alternative method of intravenous adenosine is superior to the standard method in the successful termination of PSVT.

## Methods

### Study design and setting

We conducted a pilot, parallel, open-labeled, randomized controlled trial to compare the success rate between the standard intravenous adenosine method and alternative method in the treatment of stable PSVT in the ED at Maharaj Nakorn Chiang Mai Hospital, a university-based, tertiary care hospital, between May and October 2020.

The study including verbal informed consent was approved by the Ethics Committee of the Faculty of Medicine, Chiang Mai University (No.159/2020) on May 8, 2020, and was conducted in accordance with the principles of the Declaration of Helsinki. This study was registered in the Thai Clinical Trials Registry (TCTR20200609001) on May 21, 2020. We followed the Consolidated Standards of Reporting Trials (CONSORT) extension for the Pilot and Feasibility Trials Statement. We also followed the recommendations of trial protocol regulations of the World Health Organization.

### Selection of participants

Patients ages 18 years old or more presenting to the ED with stable PSVT after failed treatment by vagal or modified vagal maneuver, were eligible and randomized. Exclusion criteria were (1) PSVT patients with successful vagal or modified vagal maneuver, (2) pregnancy, (3) history of anaphylaxis with adenosine, (4) previous randomized PSVT patients, and (5) PSVT patients with hypotension and sign of shock or alteration of consciousness (unstable PSVT).

### Methods of measurement and interventions

Patients were screened, enrolled, and received the intervention by well-trained ACLS health care professionals, emergency medicine (EM) residents, or emergency physicians following standardized procedures. In suspected PSVT, all standard care, according to ACLS, was given to the patient including a 12-lead electrocardiogram (ECG) and ECG monitoring. PSVT patients who met the eligibility criteria gave their verbal consent.

Thirty sequentially numbered, opaque, sealed envelopes (SNOSE) containing the details of the method of an intravenous adenosine double syringe technique were utilized. For two groups of treatment methods, the standard method (control group) was defined as intravenous adenosine through a right cubital vein or as proximal as heart by T-connector or stopcock. We administered adenosine 6 mg at the rate of 1–2 s and followed by 20 mL of normal saline. The other group, an alternative method (treatment group) was the same as the standard group, plus immediately elevating the right arm to 90-degrees perpendicular to a horizontal plane for ten seconds with a stopwatch.

A sequence generation with block randomization was used with variable block sizes (block sizes 2, 4) using a web-based, randomization system (www.sealedenvelope.com) with an allocation ratio of 1:1. To conceal randomization allocation, the sealed envelope was unsealed just prior to the adenosine administration. Baseline characteristics, group of treatment including rhythm strip, were recorded. According to primary and secondary outcomes occuring within one minute after adenosine was administered, patients could recognize what method they received. The processes were not unnecessarily blinded.

In case of unstable PSVT or unstable status before giving adenosine, patients were automatically withdrawn from the study. The patient was then treated with the standard of care such as electrical cardioversion, an intravenous fluid, antidysrhythmic medication.

For the patient with an unsuccessful response to our intervention, a second dose of 12 mg intravenous adenosine or electrical cardioversion was planned to rescue as appropriate means based on their hemodynamic and standard of care.

Primary and secondary outcomes occurred within one minute after the first dose of adenosine administration; therefore, cross-over did not influence our results.

### Outcome measures

The primary outcome was the successful termination of PSVT determined by ECG response as at least two-fold of the RR-interval widening or sinus rhythm conversion on the ECG strip after the first dose of adenosine. Subsequently, the secondary outcome was a sudden complication within one minute after intravenous administration including light-headedness, palpitations, chest discomfort, dyspnea, and cardiac arrest. Light-headedness is a feeling that the participant is about to faint. Palpitations are feelings or sensations that the heart is racing. Chest discomfort or chest pain is many unpleasant or uncomfortable sensations in the anterior chest [9]. Dyspnea is a feeling of not being able to breathe well. Signs of cardiac arrest are the absence of a palpable pulse and unresponsive with absent or abnormal breathing [10].

### Data analysis and sample size estimation

We based the sample size for statistical power on the primary outcome. A previous study showed the success rate of the standard adenosine method as 89.7% [11]. We estimated that an alternative treatment would increase this success rate to 95%. A need to enroll 700 patients was needed on a one-sided alpha level of 0.05 and power of 80% with 10% of incomplete data. Although SVT is a common condition in the ED, a previous study proposed a new postural modification (modified vagal maneuver) to terminate SVT without the use of adenosine [12]. Therefore, adenosine was reserved in the patient who failed to respond to the postural modification method. A pilot chart review in our setting showed about 50 patients per year were eligible. At this rate of enrolment, 700 cases would not be feasible to achieve in the available period. Therefore, we planned to conduct a pilot study. In a previous biostatistics article of a pilot study, a minimum of 12 participants per group was considered to achieve acceptable precision [13]. In addition, the data distribution approximates the standard normal distribution if the sample size is 30 or more [14]. In our study, we had two groups (12 participants per group) and planned to compensate for 20% of incomplete data. Therefore, we planned to enroll a total of 30 participants, 15 in each group for evaluating the feasibility of a single-center trial testing superiority of the method of adenosine administration for stable PSVT.

Descriptive data were presented as number, percentage, mean, standard deviation, median, and interquartile range as appropriate. Categorical data were compared using Fisher exact test. Continuous data with and without a normal distribution were compared using the t-test and the Mann–Whitney U test, respectively. Data visualization and the Shapiro–Wilk test were used to determine the normal distribution of continuous variables. Categorical data were compared by risk difference with a 95% confidence interval (CI) in an intention-to-treat analysis. The missing data, except for treatment group and outcome data, would be handled with multiple imputation methods. The Stata version 16 (Stata Corp LLC, College Station, Texas, USA) was used for statistical analysis. Statistical significance was determined by 95% CI or *p*-value < 0.05. The datasets are available from the corresponding author on reasonable request.

## Results

### Characteristics of study subjects

**Fig. 1:**
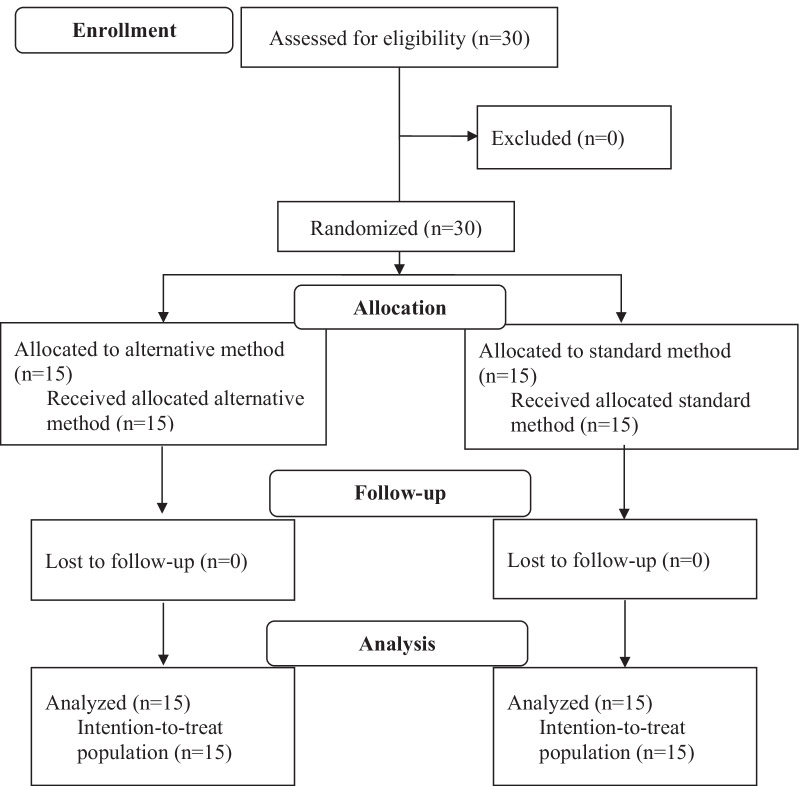
Consolidated standards of reporting trials (CONSORT) diagram of study enrollment

A total of 30 patients were eligible to enroll and randomized, 15 patients were allocated in each group. The CONSORT diagram (Fig. 1) describes the flow of patients through the trial. There were no patients excluded after enrollment in this study. All gave verbal consent and were analyzed for efficacy as intention-to-treat analysis. Baseline characteristics were comparable in both treatment groups (Table 1). Data visualization and the Shapiro–Wilk test showed the normal distribution of continuous variables. Most patients were women. Hypertension was the most common co-morbidity. Palpitations were the major chief complaint in both treatment groups.

**Table 1:** Baseline characteristics

Characteristics	Alternative method (treatment group) (n = 15)	Standard method (control group) (n = 15)	*P*-value
Age—year*	63 ± 22	55 ± 18	0.301
Female—n (%)^†^	10 (66.7)	11 (73.3)	1
Weight—kilogram*	55 ± 15	64 ± 14	0.090
*Co-morbidity—n (%)*^†^			
Hypertension	5 (33.3)	8 (53.3)	0.462
Dyslipidemia	4 (26.7)	6 (40)	0.7
Chronic kidney disease	3 (20)	4 (26.7)	1
Hypothyroidism	2 (13.3)	0	0.483
Thyrotoxicosis/hyperthyroidism	1 (6.7)	2 (13.3)	1
Atrial fibrillation	1 (6.7)	2 (13.3)	1
Valvular heart disease	1 (6.7)	1 (6.7)	1
Coronary artery disease	1 (6.7)	0	1
Diabetes	1 (6.7)	3 (20)	0.598
Heart failure	0	1 (6.7)	1
Stroke	0	1 (6.7)	1
Others	5 (33.3)	7 (46.7)	0.71
None	4 (26.7)	3 (20)	1
Alcohol drinker—n (%)^†^	4 (26.7)	2 (13.3)	0.651
Smoker—n (%)^†^	3 (20)	2 (13.3)	1
*Clinical presentation—n (%)*^†^			
Palpitation	15 (100)	12 (80)	0.224
Dyspnea	3 (20)	2 (13.3)	1
Chest pain	2 (13.3)	4 (26.7)	0.651
Syncope	1 (6.7)	0	1
Abdominal Pain	0	1 (6.7)	1
Fever	0	1 (6.7)	1
Nausea, vomiting	0	1 (6.7)	1
Diarrhea	0	1 (6.7)	1
*Current medications—n (%)*^†^			
Antihypertensive	7 (46.7)	8 (53.3)	1
Lipid lowering	4 (26.7)	6 (40)	0.7
Antidiabetic	1 (6.7)	3 (20)	0.598
Anticoagulant	2 (13.3)	1 (6.7)	1
Antiarrhythmic	1 (6.7)	5 (33.3)	0.169
Antiplatelet	1 (6.7)	0	1
Antithyroid	1 (6.7)	0	1
*Disposition from the ED—n (%)*^†^			
Discharge	14 (93.3)	7 (46.7)	0.014
Admission	1 (6.7)	8 (53.3)	
ED LOS—minute*	151 ± 64	230 ± 97	0.014

ED, emergency department; LOS, length of stay

*Data presented by mean ± standard deviation and compared using the *t*-test

^†^Data compared using Fisher exact test

### Main results

Table 2 illustrates the primary and secondary outcomes. The primary outcome, as successful termination of PSVT, produced no difference between groups (86.7% [13/15] patients in alternative method and 80% [12/15] of the patients in the standard method, risk difference 6.7%, 95% CI − 19.9 to 33.2%, *P* 1.00).

**Table 2:** Primary and secondary outcomes

Characteristics	Alternative method (treatment group) (n = 15)	Standard method (control group) (n = 15)	Risk difference (95% CI)
*Primary outcomes*			
ECG response or convert to sinus rhythm after adenosine—n (%)	13 (86.7)	12 (80)	6.7 (− 19.9 to 33.2)
*Secondary outcomes*			
Complications in one minute—n (%)			
None	14 (93.3)	14 (93.3)	0 (− 17.9 to 17.9)
Light-headedness	1 (6.7)	0	6.7 (− 6 to 19.3)
Palpitation	0	1 (6.7)	− 6.7 (− 19.3 to 6)
Chest discomfort	0	0	NA
Dyspnea	0	0	NA
Cardiac arrest	0	0	NA

ECG, electrocardiogram; NA, not applicable; 95% CI, 95% confidence interval

The complications within one minute after an intravenous administration, secondary outcomes were similar in both groups. Ninety-three percent in both groups experienced no complications. Furthermore, there was only one complication found in each group. Light-headedness in alternative treatment group and palpitations in the another. Chest discomfort, dyspnea, and cardiac arrest were the secondary outcomes with no patients experiencing these complications. There was no significant difference in secondary outcomes between groups.

## Discussion

This study was a pilot randomized controlled trial comparing the success rate of first dose intravenous adenosine between standard and alternative methods in a patient with stable PSVT. No evidence showed an alternative method to be superior to the standard method in terms of successful outcomes, for ECG response and complications within one minute after adenosine injection. According to previous studies regarding the physiology of adenosine [2, 5, 6]. The brevity of adenosine action has an extremely short half-life. (6–10 s). Then, the prolongation of RR interval is associated with this effect. Therefore, we can conclude that adenosine, in our study, proved to be a highly effective treatment for the success rate of ECG response in acute PSVT no matter how adenosine was injected.

As discussed above due to the brevity of adenosine action, the rapid uptake, and metabolism, no serious complications from adenosine administration. Only minor complications, mainly subjective symptoms; palpitation and light-headedness, which are of short duration only occurred. Our study shows no difference between those two methods, ECG response, and complications. Then, by those results, for now, adenosine administration for treatment of acute PSVT might be effective in both methods, while a standard method seems to be much easier to perform. However, that efficacy could not be concluded due to the small sample size. Further study should assess the implementation of the alternative method and data collected through the final treatment. Also, gathering all SVT patients should be considered for further study.

## Limitations

Our study had some limitations. First, since it was the pilot study, we did not find any statistical significance between groups because small sample size. To increase the number of cases, further study might be conducted as multicenter design. Second, our study was limited to only stable PSVT patients. However, unstable PSVT who eligible for intravenous adenosine were reserved to the condition that suggested SVT with aberrant conduction. This might be influenced by adenosine efficacy; therefore, further investigation should be pursued. Third, this was a study performed in a high-volume, university-based, tertiary care hospital with much experienced medical personnel to correctly perform those methods because our study attempted to standardize the ACLS health care professionals who performed the procedure. The extension of our findings to other settings, such as low incidence of PSVT or infrequently performed this procedure, should be undertaken with caution. Fourth, our outcome was an immediate response after the first of adenosine. We did not collect the final outcome in a patient who failed to terminate after the first dose of adenosine in SVT and received subsequent adenosine. Fifth, some of our secondary outcomes including light-headedness, palpitation, chest discomfort, dyspnea might be considered as subjective judgment.

## Conclusions

Both standard and alternative methods for adenosine administration in PSVT were clinically successful in terms of ECG response. Also, both methods provided no serious complications after administration. However, additional studies comparing methods through the final treatment are required to fully investigate specific treatment differences. The standard method of adenosine injection is a safe, easy-to-administer, and widely available treatment for PSVT.

## Data Availability

The datasets are available from the corresponding author on reasonable request.

## Abbreviations

ACLS: Advanced cardiovascular life support
AV node: Atrioventricular node
CONSORT: Consolidated standards of reporting trials
ECG: Electrocardiogram
ED: Emergency department
EM: Emergency medicine
LOS: Length of stay
mg: Milligram
mL: Milliliter
msec: Millisecond
PSVT: Paroxysmal supraventricular tachycardia
SNOSE: Sequentially numbered, opaque, sealed envelope
SVT: Supraventricular tachycardia
95% CI: 95% Confidence interval
